# MRG-1/MRG15 Is a Barrier for Germ Cell to Neuron Reprogramming in *Caenorhabditis elegans*

**DOI:** 10.1534/genetics.118.301674

**Published:** 2018-11-13

**Authors:** Martina Hajduskova, Gülkiz Baytek, Ena Kolundzic, Alexander Gosdschan, Marlon Kazmierczak, Andreas Ofenbauer, Maria Lena Beato del Rosal, Sergej Herzog, Nida ul Fatima, Philipp Mertins, Stefanie Seelk-Müthel, Baris Tursun

**Affiliations:** * Max Delbrück Center for Molecular Medicine in the Helmholtz Association, Berlin, Germany; † Berlin Institute for Medical Systems Biology, 13125 Berlin, Germany

**Keywords:** Automated reverse genetics, reprogramming, epigenetics, *Caenorhabditis elegans*, MRG15, germline, protein network

## Abstract

Chromatin regulators play important roles in the safeguarding of cell identities by opposing the induction of ectopic cell fates and, thereby, preventing forced conversion of cell identities by reprogramming approaches. Our knowledge of chromatin regulators acting as reprogramming barriers in living organisms needs improvement as most studies use tissue culture. We used *Caenorhabditis elegans* as an *in vivo* gene discovery model and automated solid-phase RNA interference screening, by which we identified 10 chromatin-regulating factors that protect cells against ectopic fate induction. Specifically, the chromodomain protein MRG-1 safeguards germ cells against conversion into neurons. MRG-1 is the ortholog of mammalian MRG15 (MORF-related gene on chromosome 15) and is required during germline development in *C. elegans*. However, MRG-1’s function as a barrier for germ cell reprogramming has not been revealed previously. Here, we further provide protein-protein and genome interactions of MRG-1 to characterize its molecular functions. Conserved chromatin regulators may have similar functions in higher organisms, and therefore, understanding cell fate protection in *C. elegans* may also help to facilitate reprogramming of human cells.

TO successfully reprogram cellular identities using transcription factors (TFs), the expression of genes that are usually repressed need to be activated. In some contexts, forced expression of a cell fate–inducing TF is sufficient for the activation of ectopic gene expression. One classic example is the mammalian TF MyoD, which, when misexpressed in fibroblasts, induces muscle gene expression leading to the conversion of fibroblasts into muscle cells (Davis *et al.* 1987). However, aside from fibroblasts, many cell types are less efficiently converted into muscle-like cells due to cell fate safeguarding mechanisms that prevent ectopic gene expression based on repressive epigenetic signatures [reviewed in Pasque *et al.* (2011), Gifford and Meissner (2012), Brumbaugh and Hochedlinger (2013), Becker *et al.* (2016)]. Epigenetic regulators, including histone modifiers and chromatin remodelers, as well as a variety of different factors such as kinases and RNA-binding proteins, contribute to establishing a repressive chromatin signature, and may therefore act as barriers for cellular reprogramming.

The nematode *Caenorhabditis elegans* allows *in vivo* interrogation of such regulators for their role in safeguarding cellular identities using RNA interference (RNAi)-mediated gene expression knockdown (Tursun *et al.* 2011; Kolundzic *et al.* 2018b). In contrast to knocking out a gene by mutagenesis or gene editing (CRISPR/Cas9), RNAi generally leads to a partial knockdown thereby allowing the assessment of essential genes, which cause lethality when fully depleted. We applied RNAi postembryonically to avoid early lethality, which limited a previous RNAi screen where we identified the highly conserved histone chaperone LIN-53 (CAF-1^p48^/RBBP7 in humans) as a barrier for direct reprogramming of germ cells into neurons (Tursun *et al.* 2011).

In this study, we aimed to reveal additional factors acting like LIN-53 and identified the conserved chromodomain-containing factor MRG-1 (MORF-related gene on chromosome 15 is equal to MRG15 in human) (Olgun *et al.* 2005; Takasaki *et al.* 2007) as a novel barrier for TF-induced germ cell conversion. In mammals, MRG15 is required for proliferation of neural precursor cells, regulation of premessenger RNA splicing during spermatogenesis (Chen *et al.* 2009; Iwamori *et al.* 2016), DNA repair, and protection against genotoxic stress (Hayakawa *et al.* 2010; Bleuyard *et al.* 2017). In *C. elegans*, MRG-1 plays a role in chromosome pairing, maintaining genomic integrity, repressing X-linked genes, and regulating proliferation in the germline (Fujita *et al.* 2002; Takasaki *et al.* 2007; Dombecki *et al.* 2011; Xu *et al.* 2012; Gupta *et al.* 2015). While MRG-1’s role in germline development and differentiation to produce mature germ cells are well described (Fujita *et al.* 2002; Takasaki *et al.* 2007; Dombecki *et al.* 2011; Xu *et al.* 2012; Gupta *et al.* 2015), its function in safeguarding germ cells against TF-induced conversion was unknown. Furthermore, MRG-1-interacting proteins and its genomic DNA-binding sites in *C. elegans* were not described previously. We performed an in-depth analysis of MRG-1’s interactions with proteins and DNA using immunoprecipitation combined with mass spectrometry (IP-MS) and chromatin immunoprecipitation sequencing (ChIP-seq). Interestingly, MRG-1 interacts with SET-26, which mediates repressive histone H3K9 methylation (Greer *et al.* 2014). Conversely, we found that MRG-1 associates predominantly with genomic loci carrying active histone marks, including H3K36me3 and H3K4me3. However, our study indicates that MRG-1 and SET-26 might cooperate to prevent conversion of germ cells into neurons.

Overall, understanding mechanisms that safeguard cell fates in *C. elegans* could help to identify conserved reprogramming barriers, as exemplified by the previously identified reprogramming barriers LIN-53 and FACT in *C. elegans* (Tursun *et al.* 2011; Kolundzic *et al.* 2018a), which could be targeted to facilitate the generation of tissues for future replacement therapies.

## Materials and Methods

### Worm strains

The wild-type *C. elegans* Bristol strain (N2) and strains without heat-shock constructs were maintained according to the standard protocol (Stiernagle 2006) at 20°. Transgenic lines carrying heat-shock constructs were grown at 15° unless indicated otherwise. The following strains were used in this study: BAT28 *otIs305[hsp-16.2p*::*che-1*::*3xHA*, *rol-6(su1006)] ntIs1[gcy-5p*::*gfp*, *lin-15(+)] V*, BAT29 *otIs284[hsp-16.2p*::*che-1*::*3xHA*, *rol-6(su1006)] ntIs1[gcy-5p*::*gfp*, *lin-15(+)] V*, BAT30 *otIs264[ceh-36p*::*tagRFP]*, OH3192 *ntIs1[gcy-5p*::*gfp*, *lin-15(+)] V*, BAT326 *otIs263[ceh-36p*::*tagRFP]*; *otIs305[hsp-16.2p*::*che-1*::*3xHA] ntIs1[gcy-5p*::*gfp*, *lin-15(+)] V*, BAT483 *ogt-1(ok430) III*.; *otIs305[hsp-16.2p*::*che-1*::*3xHA*, *rol-6(su1006)] ntIs1[gcy-5p*::*gfp*, *lin-15(+)] V*, BAT522 *otIs305[hsp-16.2p*::*che-1*::*3xHA*, *rol-6(su1006)] ntIs1[gcy-5p*::*gfp*, *lin-15(+)] V*; *otIs393[ift-20p*::*NLS*::*tagRFP]*, BAT527 *otIs355[rab-3p*::*NLS*::*tagRFP] IV?*; *otIs305[hsp-16.2p*::*che-1*::*3xHA*, *rol-6(su1006)] ntIs1[gcy-5p*::*gfp*, *lin-15(+)] V*; BAT606 *edIs6[unc-119p:GFP*, *rol-6(su1006)]*; *otIs305[hsp-16.2p*::*che-1*::*3xHA*, *rol-6(su1006)] V*, RB653 *ogt-1(ok430) III* [obtained from Gene Knockout project at (Oklahoma Medical Research Foundation) OMRF]; *otIs305[hsp-16.2p*::*che-1*::*3xHA*, *rol-6(su1006)] ntIs1[gcy-5p*::*gfp*, *lin-15(+)] V*; BAT32 *glp-1(ar202) III*, *ntIs1otIs305 V*, BAT1940 *sin-3(tm1276)*; *otIs305ntIs1 V*, BAT1939 *set-26(tm2467)*; *otIs305ntIs1 V*, BAT483 *ogt-1(ok430)*; *otIs305ntIs1 V*, SS104 *glp-4(bn11)*; BAT2019 *mrg-1(bar33[mrg-1*::*3xHA]) III* (CRISPR/Cas9) .

### Synchronized worm population

Synchronized worms were obtained by two standard techniques: bleaching or harvesting early hatched L1 worms. For bleaching, gravid hermaphrodites were treated with sodium hypochlorite solution as previously described (Ahringer 2006). Household bleach (5% sodium hypochlorite) was mixed with 1 M NaOH and water in the 3:2:5 ratio. Worms were washed from NGM plates with M9 buffer containing gelatin (0.05% w/v), incubated in bleaching solution for 5 min in a 1:1 ratio, vortexed, and following worm lysis, eggs were washed three times with M9 buffer. For harvesting L1 worms, plates containing shortly starved adults and freshly hatched L1 larvae were used. Worms were collected into 1.5-ml tubes by washing twice with 800 µl of M9 buffer plus gelatin. Tubes containing worms were left to stand for 2 min to allow the separation of the two stages. Adult stage worms sink faster in a solution compared to larvae because they are heavier. Within 2 min, adult worms are pelleted at the bottom of the tube, whereas L1 larvae are still swimming near the surface of the solution. The top two-thirds of M9 buffer, containing mostly larvae worms, was transferred into a fresh 1.5-ml tube and L1 larvae were collected by centrifugation at 900 × *g* for 1 min. Harvested L1 larvae or eggs obtained by bleaching were either applied directly onto RNAi plates or regular NGM plates for further maintenance of synchronized population.

### Generating the chromatin RNAi sublibrary

Candidate genes for the chromatin RNAi sublibrary were chosen based on the presence of characteristic protein domains (http://www.uniprot.org), known function in chromatin modifications and remodeling, and any direct or indirect link to chromatin function. The RNAi sublibrary was generated by compiling existing RNAi clones from the Ahringer and Vidal RNAi libraries. The list of RNAi clones in the library can be found in the Supplemental Material, Table S1. The identity of all RNAi clones was verified by sequencing. Clones that did not exist in the RNAi libraries or clones for which sequence was incorrect, were replaced by newly built RNAi clones (Table S1). Primers were designed to amplify a unique sequence for each gene of interest (preferably complementary DNA). PCR products were cloned into the L4440 vector followed by transformation into HT115 (DE3) bacteria. Resulting clones were verified by sequencing. All RNAi clones were grown on plates containing 12.5 µg/ml tetracycline (selection for presence of T7 polymerase and RNase III mutation) and 50 µg/ml carbenicillin (selection for L4440 plasmid) to ensure RNAi efficiency in future experiments. Correct RNAi clones (730 in total) were compiled into the 96-well plate format Table S1. Deep-well plates containing 1 ml of LB medium with 50 µg/ml carbenicillin per well were prepared using an automated dispensing machine (Multidrop Combi Reagent Dispenser; Thermo Scientific). Inoculated RNAi bacteria were grown by shaking overnight at 37°. Grown bacterial cultures were mixed with glycerol (13% final concentration) and stored at −80° for further use.

### RNAi screening

We used the strain BAT28 to screen for ectopic expression of the glutamatergic neuronal marker *gcy-5p*::*gfp* upon induction of the TF CHE-1. Conditions for both automated and manual RNAi have been optimized for solid media to allow precise and fast control of the right developmental stage for *che-1* misexpression. RNAi screening of the chromatin sublibrary was performed using the feeding technique, as described previously with slight modifications (Kamath *et al.* 2001). As indicated in the screen workflow in Figure S2B, we aimed to automate as many steps as possible. L1 worms were grown on solid RNAi medium in 48-well plates at 15° until the L4 stage and heat-shocked for inducing ubiquitous misexpression (Tursun *et al.* 2011) of the ASE neuron fate-inducing TF CHE-1. After 16 hr, the BioSorter + Large Particle (LP) sampler setup was used to automatically screen for ectopic *gcy-5p*::*gfp* expression. We performed the P0 screen, where synchronized L1 larvae were applied on RNAi plates, and scored adults of the same generation (P0). Standard NGM agar medium, supplemented with 50 µg/ml carbenicillin and 1 mM IPTG, was used to pour 48-well or 6-well RNAi feeding plates. The 6-well RNAi plates were dried overnight at room temperature, and then stored at 4° until use. Because the 48-well RNAi plates tend to dry out quickly, freshly poured plates were directly turned upside-down, transferred into a humid chamber (plastic box with wet paper towels) and stored at 4°. The 96-deep-well plates containing 1.2 ml of LB medium with 50 µg/ml carbenicillin per well were poured using the automated dispensing machine (Multidrop Combi Reagent Dispenser; Thermo Scientific) and then inoculated with RNAi clones of the sublibrary and grown by shaking at 37°. For the manual screen, bacteria grown for 16 hr were centrifuged for 5 min at 300 × *g*, 800 µl of the supernatant was removed, and the bacterial pellet resuspended in the remaining LB medium. Resuspended bacteria were seeded in duplicates on 6-well RNAi plates (30 µl per well) and double-strand RNA synthesis was induced overnight at 37°. The following day, synchronized worms at the L1 stage were added to RNAi plates (100–200 larvae per well) that had been precooled to 15° to avoid heat shock. To minimize the cotransfer of OP50 bacteria, worms were washed three times with M9 buffer before plating. Worms on RNAi plates were kept at 15° until they reached the L4 stage, at which time they were heat-shocked at 37° for 26 min to induce expression of CHE-1. Following heat shock, RNAi plates were shifted to 25° and scored ∼16 hr later. To check for ectopic expression of the *gcy-5p*::*gfp* reporter, we used the Olympus MVX10 and Leica M205 FA dissecting microscopes. For the automated screen, the liquid cultures of RNAi bacteria were centrifuged as described above and the majority of the supernatant was discarded by quickly inverting the 96-well plates. Bacterial pellets were resuspended by vortexing in the remaining medium, and from this suspension, 10 µl was used for seeding the 48-well plates. Seeded 48-well plates were placed under the fume hood for 1 hr to dry the bacterial lawn. Subsequently, plates were incubated in humid chamber at 37° overnight. The following day, seeded plates were cooled to 15° before applying synchronized worm populations. If necessary, seeded 48-well plates could be stored at 4° for maximum 3 days. The concentration of worm eggs or L1 larvae in M9 medium was adjusted to 100 individuals/5 µl and this volume was pipetted on each well of the RNAi plates. Worms on RNAi plates were incubated under the fume hood for 5 min to let the M9 medium be absorbed. Afterward, worms on RNAi plates were kept in a humid chamber at 15° until they reached the L4 stage. For the heat-shock treatment, 48-well plates were sealed in plastic bags and floated with the agar side up in a water bath at 37° for 8 min. After heat shock, worms on RNAi plates were placed back into the humid chamber and kept at 25° for ∼16 hr. The following day, we screened worms for ectopic *gcy-5p*::*gfp* signal using the LP sampler in combination with the BioSorter Large Particle Flow Cytometer (Union Biometrica). Before BioSorter analysis, RNAi plates were incubated at 4° for 1 hr to immobilize worms and straighten their body. This step eliminates artifacts during fluorescence acquisition caused by worm bending and clustering. The LP sampler aspirated worms from each well of the 48-well plates containing solid RNAi media. Worms were individually passed through the BioSorter system. Measurement of axial length and optical density allowed exclusion of young animals from the analysis. Worms were scored positive based on the GFP profile along the body length. Red fluorescence was used to subtract the autofluorescence background of worms. We used FlowPilot software for the BioSorter screen and data analysis. Subsequent data processing was performed using Microsoft Excel.

### Antibody staining

Antibody staining was performed using a freeze-crack protocol on whole worms (Duerr 2006; Hadwiger *et al.* 2010). After washing worms were placed between two SuperFrost Plus slides and frozen on dry ice for 30 min. Worms were cracked by quickly breaking up the slides and immersed in paraformaldehyde (PFA) or ice-cold methanol for 5 min at room temperature. After washing once in PBS, worms were incubated for 30 min in blocking solution (1× PBS, 0.25% Triton X-100, 0.2% gelatin, 0.04% NaN_3_, double-distilled H_2_O) at 25°. Primary antibody incubations were performed at 4° for 4–12 hr and secondary antibody incubations for 2 hr at room temperature. Primary and secondary antibodies were diluted in 1× PBS, 0.25% Triton X-100, 0.1% gelatin, 0.04% NaN_3_, double-distilled H_2_O. After washing off the secondary antibodies, worms were mounted on glass microscopy slides in DAPI-containing mounting media.

Histone modifications were detected with rabbit polyclonal anti-H3K27me3 antibody (catalog no. 07-449; Millipore), rabbit polyclonal anti-H3K9me3 (catalog no. ab8898; Abcam) rabbit polyclonal anti-H3K4me3 (catalog no. ab8580; Abcam), rabbit polyclonal anti-H3K9ac (catalog no. ab4441; Abcam), rabbit monoclonal anti-H3K14ac (catalog no. ab52946), and mouse monoclonal anti-H3K36me2 (a gift from Dr. Hiroshi Kimura at the Graduate School of Frontier Biosciences Osaka University). We costained with monoclonal guinea pig anti-LIN-53 (Pineda). All primary antibodies were diluted at 1:200.

Secondary antibodies were Alexa Fluor 488 goat anti-guinea pig (catalog no. A11073; Molecular Probes, Eugene, OR), Alexa Fluor 568 goat anti-rabbit (catalog no. A21069; Molecular Probes), and Alexa Fluor 488 goat anti-rabbit (catalog no. A11070; Molecular Probes). All secondary antibodies were diluted at 1:1500.

### Single-molecule fluorescence *in situ* hybridizations

Single-molecule fluorescence *in situ* hybridization (smFISH) probes against *gcy-5*, *ceh-36*, *rab-3*, *unc-119*, and *unc-10* transcripts were custom ordered from Stellaris and used according to the manual provided by Stellaris for hybridizing FISH probes. smFISH probe set sequences were as follows:

#### gcy-5:

cattcggatgctccaagaac; caattccaactcgaagcgtc; caattggaagagttccacca; tatcgcattcggtatattcc; tcccactacaacatctacat; tattggtatcagccaactgg;
tgccactcgatcaaattgga; tttacagtagtcttggtcgt; cttaaggttgcctcaacatc;
atccgcactggatatagatc; cgatcttgttaatgcctcat; tacgagctcgactctttaca;
ggaccactaattgcgcataa; ccaatactcctcattgtcaa; tctttcccaaacttgtttgt;
tggagttagtccatttgcaa; ctactgtgaatgactcccaa; atttctaacagcatccgcaa;
tgccatcccgtataagtaaa; tagtaaccatttgcggcata; gcggtagagattttgaccaa;
tcatgttaactagtgccact; ccgtgacaattgcgaagacg; cgtttttctttttgtggcat;
gtgatcttcgactatttggc; actttctccggttatagttg; gctatgatgtttggtggtta;
atttctccttctcttcttta; ggtccatcgatagataatcc; gatatcctgaagtgatcctc;
aaagttcataccctctgcaa; ggcaagtagctgaacgtaga; ctcccaatccaaaatctgtt;
tacgatttcctccttttttc; aagtattaactccggtcgga; acttgcaaatttgctcagct;
gttctgcaacttgttttgga; tctccaattgattccacttt; tgtcggtaacccagaaacac;
ggaaccttgaagctcttaca; gcccactattaattccaatt; atggatagaccaacgacacc;
gtatccccaaataggcaata; tttccattactttccattct; tgtgcagcttctgacatatg;
tctcctcttgaacttgtttc; tgtttccattacaccttttc; gattttgtgtcactgtcagt;

#### ceh-36:

gtgtagaagttggtggtcat; ggataagcagtgtagccgag; tgcggcagcaaatgcaaatt;
atgtaagactgggtgccgtg; cattgtcgttgagcttgtgg; ttcactgtttggagccattg;
ctctgttgaacgaggtacgt; ttttccagctgatcgagttg; gatactgtgtttcgcggaaa;
gcttctcttctgtgcacatc; caaattgattgccttcgcca; ttacttgtacccttccatca;
cgatttttgaaccaaaccgt; gttgtttctatccttggctc; gatggactccatccattttt;
gatcttgatgaagtgcttcc; cgttgtgtggagaaccattg; gtgatttagtatcaggcttt;
tgtgcctggtatgtgaattc; cactgtgtgcattgaattcc; gagtttgcctcatatttggc;
ttgcagttgactcaagactg; agtcctccagttcacttttt; atttggtatctgcaagtggt;
cttgagcctgaggaagaagt; agttgcgtaggatgcatatg; tagttgtacgggtaaggagc;
gtttgatgggaagtagctgt; tgcttccatattgttggtag; aggcagtaatatttggggtg;

#### rab-3:

caaagttctgatcgggttgt; atcaggagcttgaacatgta; tccaactgatgaatttccga;
catcacagtaacggaagagg; gtagagacgaaggcagaagt; cactttgaaatcgattccga;
tttgtctccacggaacacag; ggtatcccagatttgaagtt; gatagtaggcggtggtgatg;
cagaatgaatcccattgctc; actcttcattagtgatgtca;gcaccaatcctgaacactat;
tttcccatgagtatgtcttg; ccaaccaaaacaacttgagc; ttcagagtccatatcacatt;
ccctatccatagatacaact; aagttgatcagcaagttggc; ggctgatgtttcgaagaatt;
cctttacattaatgttctcc; tctccaccaacttctcaaaa; tctgccatcttatcacaaat;
ctgtgggtccttatccaaac; ttcgagcttctgtccttttg; aattgcattgctgttgagca;
attgcgtttggaatttggga; agagctacgcgcttttagaa; cctagatgttgagagaggga;
tttacgatccatatatctgg; taattaaaccaactacgccc; ggggaatatgattgaacgtt;
gctctgggaattgtttggaa; ggcgactatgattagttaga; tgggaactgggaagtcacta;
aatcaatctttcagcgggtg; cctcgaaaataatttcctcc.

#### unc-10:

taaatccggcatcatcgacg; ttcacgttcttctgcagata; ccgtgatctgtttgtctaac;
agatttgacagatcgcgtca; caattccgtccgcaaatttg; cagattgccttatttttgct;
gattttgactcattggctgt; catattctgattgtggctct; tttgtcctttgttggttttg;
aggcgtttgtttcatagttc; tcttgttgtccatgttgatt; attctctctcattctgttgt;
tctcggaatttccagtgtag; ggttgttttggttctgattc; ggttcaaatggtcgtcagta;
tcgaagttgcctatgcaatc; cgagtttttatgatcgccat; atggtgacaaggacagcgat;
ccctgttccaaaatgatcat; tggctgcagaattttcagtt; gtaatgaatgcaccgagctt;
atgtggcattttgcagagac; ttgcagcgatgctatcatat; gaatacgccgaggatgacat;
gatggatatgcagatggcac; ggctgattgtgaatgtggta; gatgtcgaacgattgcgtga;
gagcaactgagagttgtcga; aaactggcatgagtgtctct; ggttcagtaaggccattata;
tcgtaatcccagacagttag; gtcatttggggcaagatgat; tcgtcgtcgtcaatgtattc;
atgatcagatgtgtagcctg; tgttggatcgtacatatcct; tccatcactataatatccct;
cattgtagttggcatgctat; aaacttttctttcgctcctt; cctcagatctagcaaaaccg;
gtgagccgatctgaagacaa; cttgcttcagaaagggagga; gcaaacttgtctgagtgagc;
aagcacttgacgaccgacaa; cttttacatagggagctgga; tttggcaatgcattgtttgc;
ttccatacgaccgtaatcac; tttgcgaaatccccatgaat; cagtttataccaccctatta;

#### unc-119:

cgatcgattgttgttgttgc; catctgagacgggaaggttg; gttatagcctgttcggttac;
tgatttttcgcgagaagctc; agagctagcacatcatttgg; gcataggaatccttgagtga;
ttatagacgtttgccgatgg; cgaggtcacggatttggaat; gcaatttcgaagagcacgtg;
attctcttccgtctcatttt; gatatcggacatatcttgcc; aatgtgtgatcggcacatcg;
aagtgccgttcaatcattcg; gcatttcaataaacgatcct; ggcatacagaatccaaattc;
tgttcacagttgtttctcga; gttgttgtgaaagttgtgga; attattgatcatgtcgtcca;
aatagaagctatcggagcgg; gtgcattacgagcttattct; tgcatcatacgagtagtcgg;

### Western blot

Control and *mrg-1* RNAi-treated worms were washed off, collected in SDS/PAGE sample buffer, and frozen at −20°. Immediately before loading, samples were boiled for 10 min and centrifuged. Histone modifications were detected with rabbit polyclonal anti-H3K27me3 antibody (catalog no. 07-449; Millipore) at a dilution of 1:1000, rabbit polyclonal anti-H3K9me3 (catalog no. ab8898; Abcam) at 1:1000, rabbit polyclonal anti-H3K4me3 (catalog no. ab8580; Abcam) at 1:1000, rabbit polyclonal anti-H3K9ac (catalog no. ab4441; Abcam) at 1:500, and rabbit monoclonal anti-H3K14ac (catalog no. ab52946) at 1:2000.

As a standard loading control, we used the rabbit polyclonal anti-histone 3 (catalog no. ab1791; Abcam) at 1:5000 dilution, and the secondary anti-mouse HRP antibody (catalog no. sc-2005; Santa Cruz Biotechnology) at 1:5.000 dilution or anti-rabbit HRP antibody (catalog no. sc-2357; Santa Cruz Biotechnology). The Lumi Light detection kit (Roche) and the ImageQuant LAS4000 system (GE Healthcare Life Sciences) were used for the signal detection.

#### Generation of CRISPR alleles:

CRISPR engineering was performed by microinjection using a PCR repair template containing the 3xHA tag sequence. The injection mix contained Cas9 protein (0.3 mg/µl), as well as a CRISPR RNA targeting *mrg-1* (100 ng/µl). Overall, we used a recently described procedure (Dokshin *et al.* 2018). CRISPR RNA sequences are: 5′GGATCTCTCGCCGCCGACGA3′, 5′GTTCGCTCCAACTCCGTCGT3′.

### IP-MS

Each immunoprecipitation was performed in triplicate. L4-stage wild-type and *mrg-1*::*3xHA^CRISPR^* worms were collected by M9 buffer, washed four times with M9 to get rid of bacteria, and concentrated into worm pellet after the last wash. The worms were added into liquid nitrogen drop by drop, by ensuring that the resulting “worm beads” did not exceed size of a black pepper to achieve even grinding afterward. The frozen worms were then cryo-fractured using a pulverizer. To obtain a fine powder, worms were further ground using a mortar and pestle on dry ice. The worm powder was resuspended in 1.5× of lysis buffer (20 mM HEPES pH 7.4, 150 mM NaCl, 2 mM MgCl_2_, 0.1% Tween 20 and protease inhibitors), dounced with tight douncer 30 times and sonicated using a Biorupter (six times 30 sec ON, 30 sec OFF; high settings) followed by centrifugation at 16,000 × *g* at 4° for 10 min. The supernatant was removed to 2 ml Eppendorf tubes and incubated with following antibodies: N2 lysates with anti-MRG-1 (Novus) or with preimmune serum for control samples; *mrg-1*::*3xHA^CRISPR^* and N2 lysates (negative control) with HA antibodies (Roche) for 30 min on a rotator at 4°. Next, µMACS ProteinA beads (Miltenyi Biotec) were added into samples as instructed in the kit and samples were incubated for 30 min at 4° rotating. Meanwhile, the µMACS columns were placed to magnetic separator to be equilibrated and ready for sample application. Samples were diluted 5× of their volume with lysis buffer before being applied to columns and the columns with bound proteins were washed three times with lysis buffer to remove background binders. The proteins were eluted with elution buffer (100 mM Tris-Cl pH 6.8, 4% SDS, 20 mM DTT), heated to 95°. Eluted samples were prepared for mass spectrometry measurements by SP3 (Hughes *et al.* 2014), before analyzing on a Q Exactive Plus (Thermo Scientific) connected to a Proxeon HPLC system (Thermo Scientific). Label-free quantification was performed using MaxQuant as described below.

### IP-MS analysis

The raw mass spectrometry data were first analyzed using MaxQuant Software (Cox and Mann, 2008) and the resulting “proteinGroups.txt” was then processed using the Bioconductor R package DEP v1.0.1 following the section “Differential analysis” of the vignette (https://bioconductor.org/packages/release/bioc/vignettes/DEP/inst/doc/DEP.html#differential-analysis; version from November 17, 2017) with minor adjustments. First, we set the random seed to the number 123 to receive reproducible results and then we followed the paragraphs on “Loading of the Data” and “Data Preparation” of the vignette to create our raw protein table. Then we extracted the associated UniProt IDs from the raw protein table and queried them on the UniProt ID mapping tool (http://www.uniprot.org/uploadlists/) to generate a mapping from “UniProtKB AC/ID” to “Gene name,” which was downloaded as a mapping table in tsv format. The unmapped IDs were manually curated by a search in the UniProt Knowledgebase (UniProtKB) and then appended to the mapping table. This mapping table was loaded into R, where we first removed all rows of the table containing duplicated UniProt IDs. Next we created unique gene names by appending to each duplicated gene name its number of occurrence separated by a dot, then we merged the raw protein table with the mapping table based on ID and UniProt ID, respectively, while keeping all rows of the raw protein table, and updated those entries in the names column where a gene name was available in the mapping table. Next, we loaded the table specifying the experimental design of the IP-MS analysis followed the instructions of the paragraph “Generate a SummarizedExperiment object” and “Filter on missing values,” where we decided to perform the less stringent filtering approach to keep those proteins that are identified in two out of three replicates of at least one condition. Then, we performed the steps described in “Normalization” and imputed the missing data using random draws from a manually defined left-shifted Gaussian distribution, with a shift of 1.8 and a scale of 0.3 as proposed in the “Impute data for missing values” paragraph. Next, we followed the paragraph “Differential enrichment analysis” to identify proteins being significantly enriched in comparison to the control co-immunoprecipitation with a minimum log2 fold change of 2 plus *t*-test with an adjusted *P*-value (α) < 0.05 (see Table S5).

### ChIP-seq

The ChIP experiment was carried out as previously described (Seelk *et al.* 2016). In brief, worms [wild type and *glp-4(bn2)*] at L4 stage were washed off plates using M9 buffer and flash-frozen as “worm popcorn” in liquid nitrogen. The popcorn was pulverized using a biopulverizer before further grinding to a fine powder using a mortar. The powder was dissolved in 10 vol 1.1% formaldehyde in PBS plus 1 mM PMSF, and fixed for 10 min with gentle rocking. Quenching was achieved by adding 2.5 M glycine to a final concentration of 125 mM and gently rocking for 5 min. After centrifugation the pellet was washed with ice-cold PBS plus 1 mM PMSF, before it was resuspended in FA buffer (50 mM HEPES/KOH pH 7.5, 1 mM EDTA, 1% Triton X-100, 0.1% sodium deoxycholate, 150 mM NaCl) plus 1% sarkosyl and protease inhibitor, and sonicated twice using a Bioruptor (15 times, 15 sec ON, 15 sec OFF; high settings) followed by 15 min centrifugation at full speed, at 4° . The supernatant was taken off (∼2–4 mg protein) and incubated either with MRG-1 antibody (Novus) or with buffer ON at 4° on a rotator. Next, samples were incubated with µMACS ProteinA beads (Miltenyi Biotec) for 1 hr on ice before they were applied to µMACS magnetic M columns that were equilibrated using FA buffer. The columns with bound material were washed 2× using FA buffer followed by washing with FA buffer plus 1 mM NaCl and FA buffer plus 500 mM NaCl. After further washing with TEL buffer (0.25 mM LiCl, 1% sodium deoxycholate, 1 mM EDTA) and 2× with TE buffer, the samples were eluted using elution buffer (1% SDS, 250 mM NaCl, 10 mM Tris pH 8.0, 1 mM EDTA). The fixation was reverse crosslinked using 2 µl of 10 mg/ml Proteinase K at 50° for 1 hr followed by incubation at 65° ON. The DNA was purified using the QIAquick PCR purification kit in a final volume of 40 µl. The DNA concentration was measured using Qubit dsDNA HS assay kit and libraries were prepared using the NEXTflex qCHIP-Seq v2 kit according to manufacturer’s instructions. After measuring the DNA quality using Bioanalyzer DNA1000 kit and Qubit dsDNA HS assay, sequencing was carried out at a HighSeq4000 as paired-end sequencing 2× 75 bp.

### ChIP-seq analysis

#### Alignment:

ChIP-seq reads were mapped using bowtie2 v2.3.2 (Langmead and Salzberg 2012) in paired-end mode with default settings (-D 15 -R 2 -N 0 -L 22 -i S,1,1.15) and allowing up to one alignment per read (-k 1) to version ce10 of the worm genome. Resulting alignment files were converted from SAM to BAM format, and indexed using samtools v1.5. Additional BigWig tracks were generated from the alignment files using bedtools bamtobed v2.25.0 (Quinlan and Hall 2010) and an in-house R script.

#### Peak calling and differential analysis:

Peaks were called for each replicate of both conditions (“glp-4,” a wild type) using the MACS v2.1.0.20151222 (Zhang *et al.* 2008) module callpeak with genome size set to the worm genome, skipping of model building process and extension of reads in 5′–3′ direction to 300 bp (-g ce -keep-dup auto -q 0.05 - nomodel -extsize 300).

The resulting peaks were than analyzed using the Bioconductor R package DiffBind v2.66 following the section “Example: Obtaining differentially bound sites” of its vignette (http://bioconductor.org/packages/release/bioc/vignettes/DiffBind/inst/doc/DiffBind.pdf; edited March 27, 2017; compiled January 19, 2018). Within R we defined a sample sheet with a similar structure as the example and used the dba() function to load all peaks, count the total number of unique peaks after merging overlapping ones (8226) and the total number of peaks that overlap in at least two of the samples (6723). Then, we calculated a binding matrix with scores based on read counts for every sample using the dba.count() function with the summit argument set to 250, leading to centering of the peaks at their point of highest enrichment (summit) and extending them 250 bp upstream and downstream from there. Next we established the contrast based on the tissue metadata, and performed the differential analysis to detect significantly differentially bound peaks with a minimum fold change of 1 and a maximum adjusted *P*-value of 0.05 (1183).

#### Gene annotation:

The peaks identified by MACS were annotated by overlapping with the ENSEMBL assembly annotation WBcel215 version 70. The genes in Tables S4 and S5 were defined by deriving the set of unique gene names of all overlapping genes.

#### Correlation with histone modifications:

Wiggle signal data files created from ChIP-seq experiments were downloaded for a selection of histone modifications that shared experimental conditions, from modEncode database (Gerstein *et al.* 2010). The tool CrossMap v0.2.1 (Zhao *et al.* 2013) was used to perform a liftover of the wiggle tracks from assembly ce6 to assembly ce10 and exporting to BigWig format on the fly. The module multiBigwigSummary of the software deepTools v2.5.1 (Ramírez *et al.* 2016) was used to calculate the average score for equally sized bins of 10 kb size, covering the whole genome for all BigWig tracks of the histone modifications and the BigWig tracks of the wild-type MRG-1 ChIP-seq alignment. The resulting matrix was then used to calculate the Spearman correlation between the histone modifications and the binding profile of MRG-1 (Figure S7A).

#### MRG-1 ChIP-seq peak heatmaps:

The heatmaps were prepared and plotted using the Bioconductor R package genomation v1.10.0 (Akalin *et al.* 2015). To define the rows of our heatmap, we took the summit centered peaks, fixed the center point, and extended them to a total length of 4000 bp. Then we used the function ScoreMatrixList() from genomation to create a matrix (ScoreMatrix) for every sample, where we have *m* rows, for *m* being the number of peaks, and *n* columns, for *n* = 50. The columns are constructed by subsetting every peak into n bins of equal width and calculating the average read-count per million per bin from the respective BAM files of the sample. Because the scores per row can have a high dynamic range, it is sometimes convenient to scale the matrix before plotting, so we scale and center each matrix using the function scaleScoreMatrixList(). This procedure was performed for all peaks that overlap in at least two of the samples (6723), for all significantly differentially bound ChIP-seq peaks between N2 and *glp-4(bn2)* background with a false discovery rate < 0.05 (1183), and for all differentially bound ChIP-seq peaks between N2 and *glp-4(bn2)* background with a false discovery rate < 0.05 and fold change > 2 (409), to create Figure S6, A–C.

#### Histone modifications peak heatmap:

For the peak heatmaps of the modEncode-based histone modifications, we created a ScoreMatrixList (explained above) with *n* = 400 bins with the score calculated from the BigWig signal tracks. The “soma + germline” heatmap was based on all peaks that overlap in at least two of the N2 samples (5141) and the “germline-specific” heatmap is based on all those peaks which were only identified in N2 background but not in *glp-4(bn2)* background (521).

#### Meta-region profile:

The meta-region profile of the modEncode-based histone modifications was acquired by plotting the ScoreMatrixList in a histogram representation to show the column-wise average of centered and scaled ScoreMatrix. The heat map of the meat region profile represents a set of meta region profiles as a stack of heatmaps.

#### Genome Browser shot:

The Genome Browser view shows the region “chr10:117,922,301-119,587,630” of the ce10 genome assembly and was created using the Bioconductor R package Gviz (Hahne and Ivanek 2016).

### Microscopy

Worms were mounted on freshly made 2% agarose pads for fluorescence and Nomarski imaging. We used 10 mM tetramizole hydrochloride (2,3,3,6 tetrahydro-6-phenylimidasol) in M9 buffer to anesthetize animals. Microscopy analyses were performed using the Axio Imager.M2 (Zeiss, Thornwood, NY) equipped with a sensitive charge-coupled device camera (Sensicam qe; PCO Imaging). MicroManager was used for image acquisition and processing (Edelstein *et al.* 2010, 2014).

### Data availability

ChIP-seq records are available at https://www.ncbi.nlm.nih.gov/geo/query/acc.cgi?acc=GSE110969. Supplemental material available at Figshare: https://doi.org/10.6084/m9.figshare.7303859.

## Results

### Setup for automated chromatin RNAi sublibrary screening

**Figure 1:**
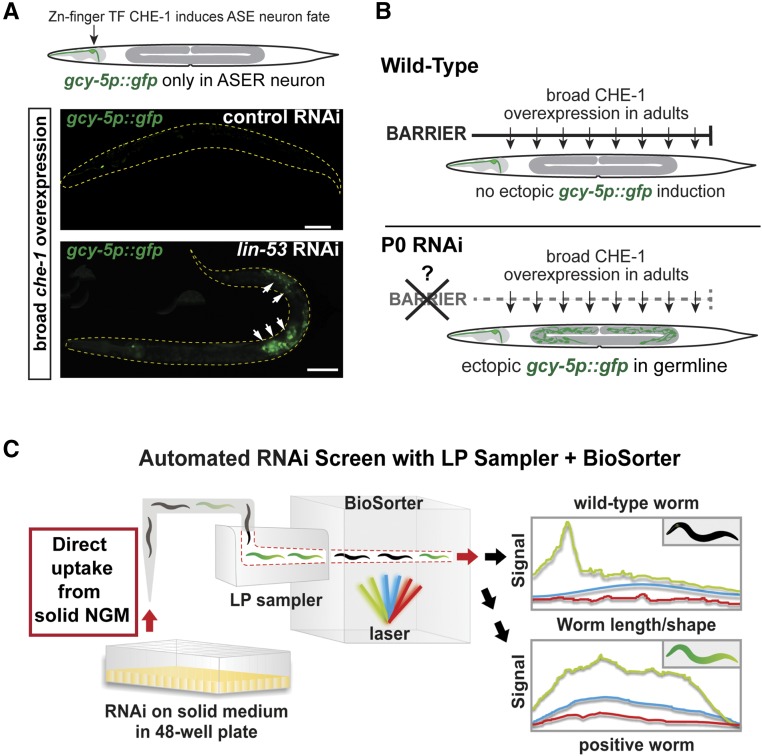
Automated RNAi screen for epigenetic reprogramming barriers. (A) Misexpression of CHE-1 and F1 RNAi against the histone chaperone gene *lin-53* (Caf1p48/RBBP4/7) on solid worm growth media allows germ cell conversion (GeCo) to ASE neuron-like cells as visualized by *gcy-5p*::*gfp* expression in the germline (white arrows). Bar, 50 μm. (B) Ubiquitously misexpressed TF CHE-1 is blocked by reprogramming barriers to induce the glutamatergic ASE neuron fate visualized by the ASE-specific reporter *gcy-5p*::*gfp* in P0 RNAi screening to identify epigenetic barrier factors, which block germ cell conversion. (C) A solid media-based automated RNAi screening system by combining the BioSorter with a robotic large-particle sampling system (LP sampler, both Union Biometrica). The LP sampler collects worms from solid RNAi medium by repeated flushing and aspiration and directly transfers worms to the BioSorter for fluorescence-intensity scanning. Detailed analysis of aspiration and sorting efficiency is shown in Table S2. Bar, 20 μm.

To perform RNAi screens for chromatin factors that safeguard cell fates, we used a previously described transgenic strain carrying the *gcy-5p*::*gfp* reporter, which specifically labels the ASER neuron, and the *hsp*::*che-1* (heat-shock promoter controlled *che-1*) transgene that allows broad misexpression of the TF CHE-1. CHE-1 induces the fate of specific neurons termed ASER/L, but its broad overexpression does not lead to reprogramming of other cells in wild-type or control backgrounds (Figure 1A). However, RNAi against the histone chaperone gene *lin-53* allows germ cell reprogramming to ASE neurons upon *che-1* overexpression, as previously described (Figure 1A) (Tursun *et al.* 2011; Kolundzic *et al.* 2018a,b). We aimed to screen for more factors that prevent *che-1*–induced reprogramming by exposing animals to RNAi only after embryonic development (P0 RNAi) (Figure 1A). This strategy allows the assessment of factors that cause embryonic lethality, or developmental arrest, when animals are treated with RNAi during embryogenesis by exposing their mothers to RNAi (F1 RNAi). We generated an RNAi sublibrary targeting all known factors (∼800) that have been implicated in chromatin regulation (Cui and Han 2007; Lai and Wade 2011; Shaye and Greenwald 2011; Wenzel *et al.* 2011), including a variety of different protein families (Figure S1A and Table S1). Since germ cell reprogramming efficiency drops significantly in liquid RNAi compared to solid media RNAi (Figure S1B), we had to establish a solid phase–based RNAi screening pipeline by combining an LP sorter (BioSorter) with an automated sampling system (LP sampler) (Figure 1C and Figure S2). A previously described automated RNAi screening procedure from solid media requires manual transfer of worms to the sorting unit (Squiban *et al.* 2012). In contrast, the new setup allows a fully automated transfer of worms, which are then automatically analyzed for changes in the pattern of fluorescence (∼20 worms/sec) (Figure S2 and Table S2). The high sensitivity of this system allows for the detection of increased GFP derived from only one additional cell (Figure S2), thereby making it a sensitive and powerful tool to screen for factors that block induction of ectopic GFP expression.

### The chromodomain protein MRG-1 is a barrier for germ cell reprogramming

**Figure 2:**
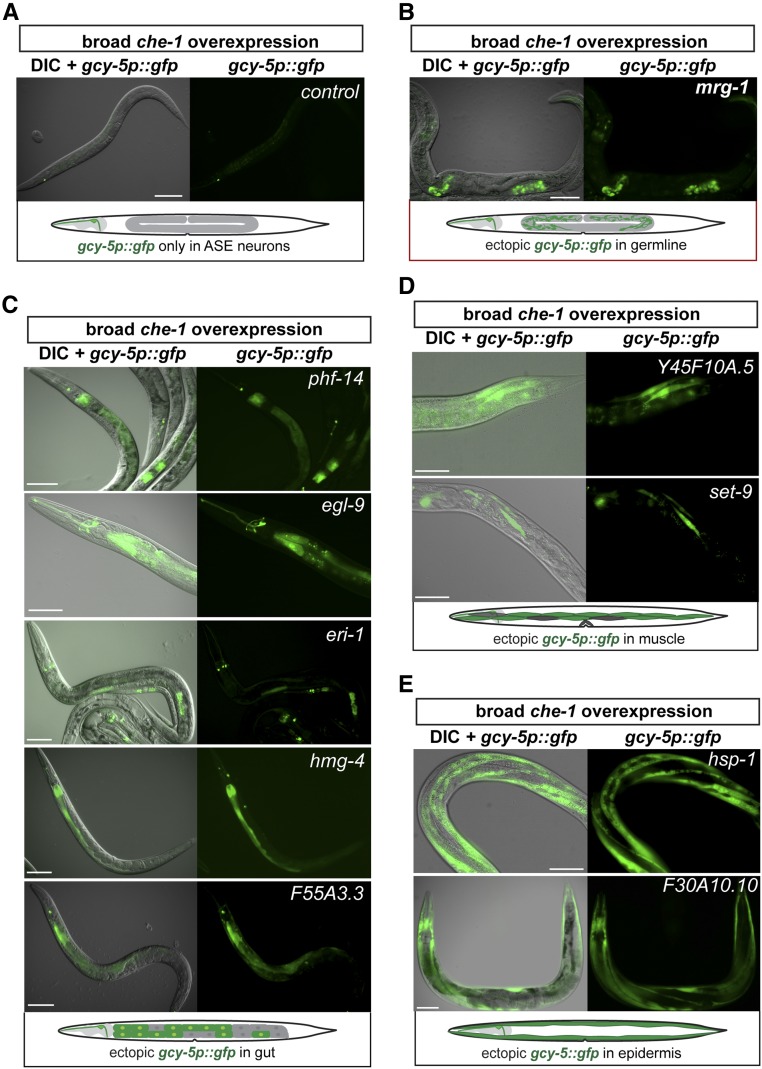
Ectopic expression of *gcy-5p*::*gfp* observed in an automated P0 RNAi screen. (A) Transgenic background *hsp*::*che-1*, *gcy-5p*::*gfp* used for screening. RNAi control worms show expression of *gcy-5* only in the ASER head neuron. (B–E) Ectopic induction of *gcy-5p*::*gfp* is detectable in different tissues including germline (B), gut (C), muscle (D), and epidermis (E) depending on the RNAi target. Only depletion of *mrg-1* encoding a chromodomain-containing protein (orthologous to human Mortality factor 4-like protein 1/MRG15) shows ectopic *gcy-5p*::*gfp* in the germline. Bar, 20 μm.

**Figure 3:**
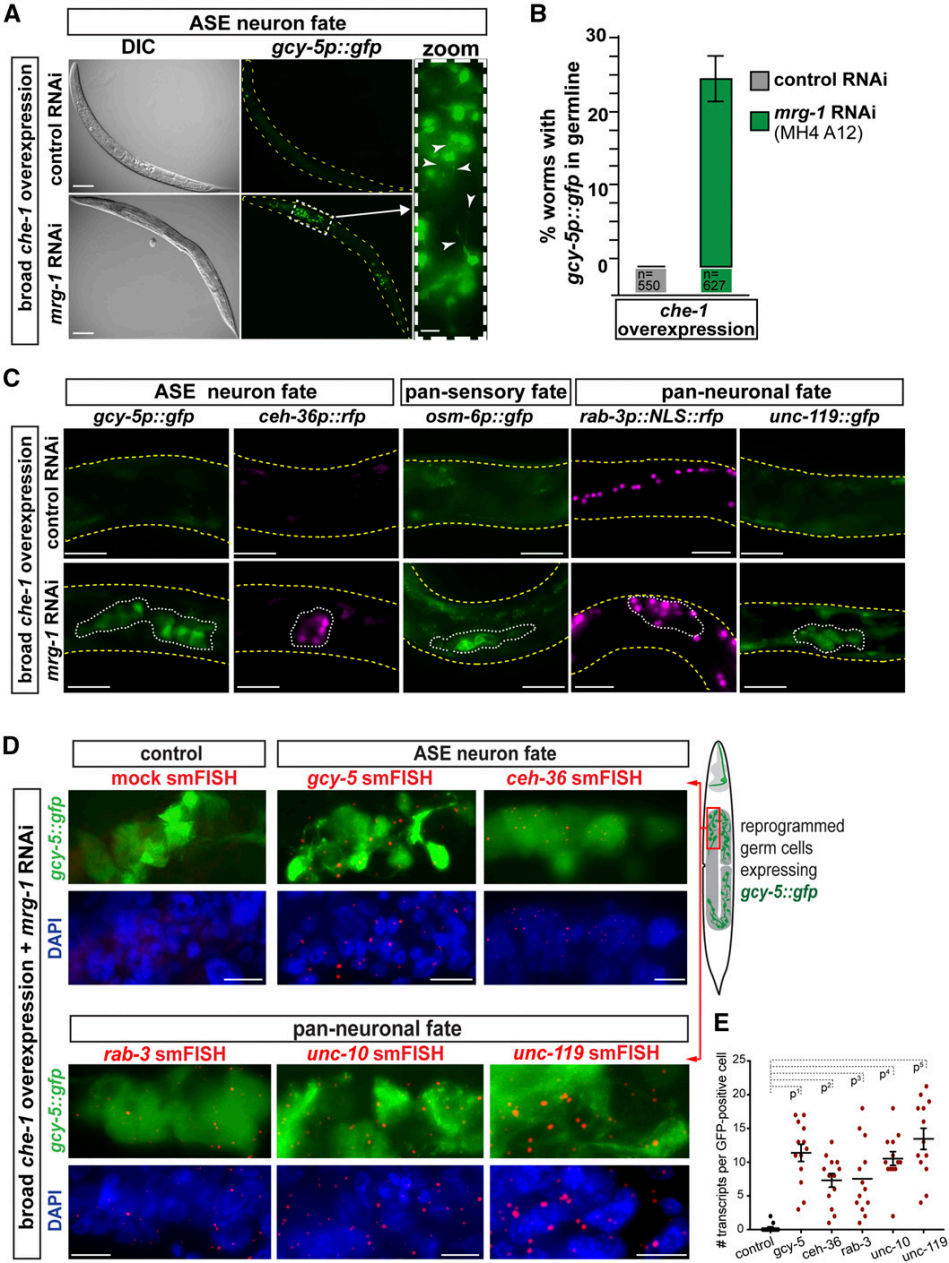
RNAi against *mrg-1* results in conversion of germ cells into neurons. (A) RNAi control animals show *gcy-5p*::*gfp* expression only in head neurons. RNAi against *mrg-1* allows misexpressed *che-1* to induce *gcy-5p*::*gfp* expression in the germline. Magnification (white dashed box) reveals that GFP-positive cells adopt neuronal morphology by showing axo-dendritic outgrowths and protrusions (white arrow heads). Bar, 20 and 5 μm. (B) Quantification of animals that show GFP in the germline when treated with *mrg-1* RNAi and *che-1* mis-expression. Number of animals (*n*) quantified are indicated. Error bars represent SEM. (C) RNAi against *mrg-1* allows *che-1* to induce expression of additional neuronal gene reporters. *ceh-36p*::*rfp* is specific for glutamatergic ASE and AWC neurons, *osm-6p*::*gfp* is specific to pan-sensory neurons such as ASE, and *rab-3p*::*nls*::*rfp* and *unc-119p*::*gfp* are pan-neuronally expressed genes. White lines outline areas of the germline with GeCo. Yellow lines outline worm body. Bar, 10 μm. (D) smFISH to detect transcripts derived from endogenous neuronal genes in GFP-positive (*gcy-5p*::*gfp*) germ cells. Messenger RNA molecules are visible as red dots. Control was incubated with mock hybridization. Bar, 2 μm. (E) Quantification of smFISH detections based on counts of hybridization signals (red dots) per GFP-positive cells. For each condition, 20 GFP-positive cells were counted for smFISH-derived transcript detection based on fluorescence signals as exemplified in D. *P*-values based on ANOVA with Dunnett’s multiple comparison test: *P*^1^ = 0.0001, *P*^2^ = 0.0003, *P*^3^ = 0.0002, *P*^4^ = 0.0001, *P*^5^ = 0.0001.

By performing a P0 RNAi screen to identify factors that prevent germ cell to neuron conversion, in combination with the BioSorter, we detected increased GFP expression derived from the *gcy-5p*::*gfp* transgene upon RNAi against 10 target genes (Figure 2, A–E). Depletion of different target factors create permissiveness for *gcy-5p*::*gfp* induction by CHE-1 in distinct tissues such as the intestine and epidermis (Figure 2, A–E). We focused on the target *mrg-1* because closer examination revealed that RNAi against *mrg-1* yields a phenotype resembling the germ cell-to-neuron conversion (Figure 2B), as seen for *lin-53* F1 RNAi (Figure 1A). MRG-1 is orthologous to the mammalian chromodomain-containing MRG15, a component of the NuA4 histone acetyltransferase complex (Chen *et al.* 2009), and has recently been shown to regulate the differentiation of germ cells in *C. elegans* (Gupta *et al.* 2015). Assessment of the ectopic *gcy-5p*::*gfp* induction in *mrg-1* RNAi animals revealed that germ cells undergo conversion into neuron-like cells (Figure 3, A and B), as previously observed when targeting the Polycomb Repressive Complex 2 (PRC2) genes, including *lin-53*, by RNAi (Patel *et al.* 2012).

RNAi against *mrg-1* without overexpressing *che-1* does not cause any ectopic *gcy-5p*::*gfp* induction or loss of germ cell characteristics (Figure S3, A and B), which excludes the possibility that germ cells converted due to teratoma formation, as previously described (Ciosk *et al.* 2006). The converted germ cells show morphological changes with neuronal characteristics, including projection-like extensions (Figure 3A), and start expressing neuron subtype-specific as well as pan-neuronal marker genes, such as *ceh-36* (ASE/AWC glutamatergic), *osm-6* (pan-sensory), *rab-3* (pan-neuronal), and *unc-119* (pan-neuronal) (Figure 3C) (Tursun *et al.* 2011; Patel *et al.* 2012). To assess whether the neuronal reporter transgenes reflect expression of transcripts derived from endogenous genes, we performed smFISH (Figure 3, D and E and Figure S3, B–D). smFISH revealed that GFP-positive germ cells turn on endogenous expression of *gcy-5*, *ceh-36*, *rab-3*, the pan-neuronal RIM homolog *unc-10*, and *unc-119*, with levels comparable to authentic neurons (Figure 3, D and E and Figure S3, B–D). Endogenous expression of these neuronal genes further corroborates that germ cells faithfully convert to neuron-like cells.

### Converted germ cells upon *mrg-1* RNAi lose germline characteristics

**Figure 4:**
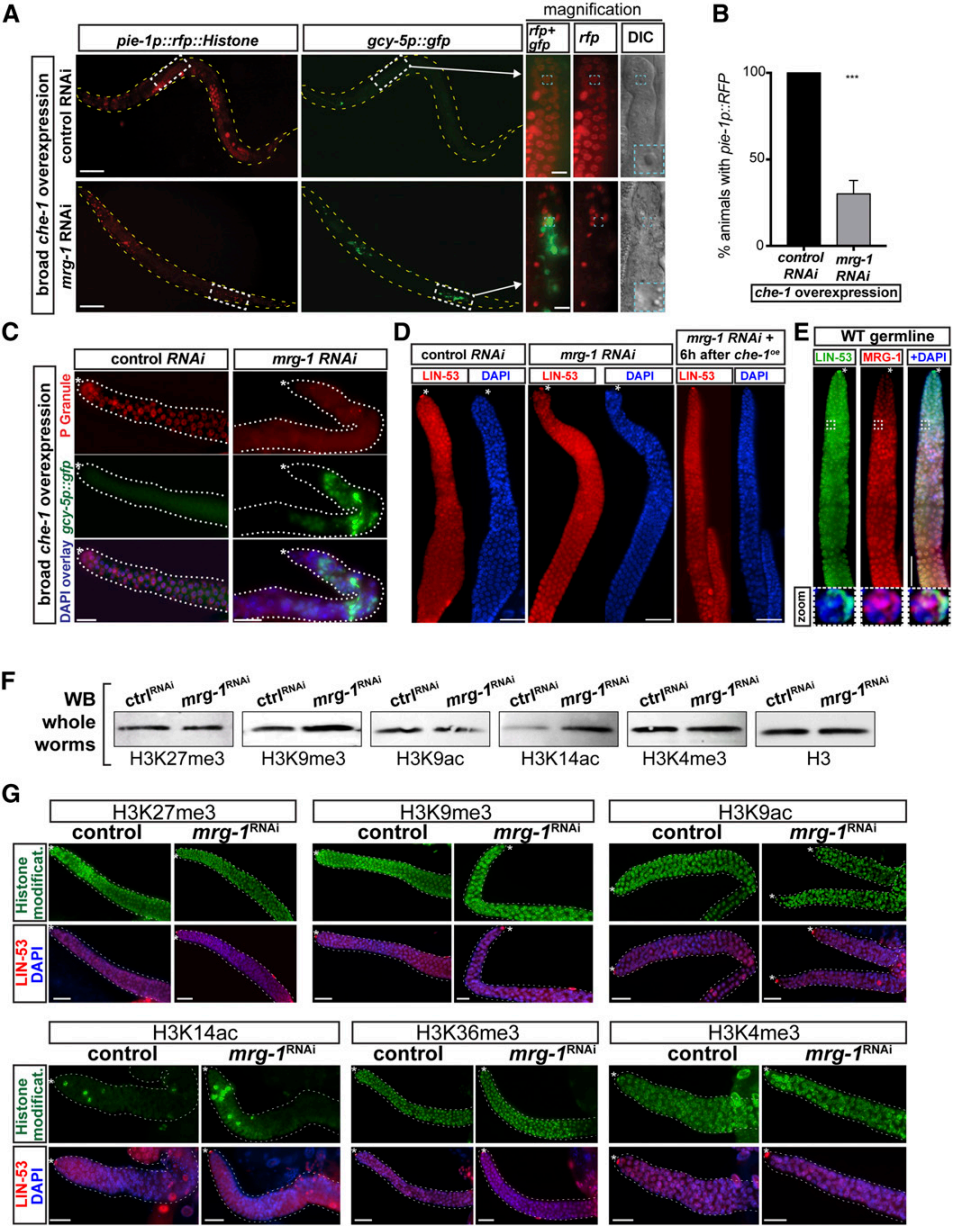
Changes of germline fate and histone modifications upon *mrg-1* RNAi. (A) Induction of *gcy-5p*::*gfp* in *mrg-1* RNAi-treated worms leads to loss of germ cell characteristics. Expression of the germline-specific reporter *pie-1p*::*rfp* diminishes in cell with ectopic *gcy-5p*::*gfp* expression as revealed upon magnification of the germline area with germ cell conversion (white dashed box). The blue dashed boxes highlight a control and a reprogrammed germ cell that has lost the typical fried egg-shaped appearance of the nucleus and now displays a rather speckled nucleus typical for neurons. Bar, 50 μm (5 μm magnification). (B) Quantification of *pie-1p*::*RFP* reporter loss in reprogrammed germ cells upon *mrg-1* RNAi; 150 animals in triplicate experiments were assessed. Error bars represent SEM. P value based on T-test = < 0.05 (C) Antibody staining for germline-specific P granules upon *mrg-1* RNAi-mediated conversion of germ cells to ASE neuron-like cells. Bar, 5 μm. (D) LIN-53 antibody immunostaining of young adult hermaphrodite germlines from control and *mrg-1* RNAi-treated animals with and without *che-1* overexpression. Asterisk indicates distal tip of the gonad. Bar, 5 μm. (E) Antibody staining of MRG-1 and LIN-53 proteins in the distal wild-type germline of a young adult hermaphrodite. The magnified germ cell nucleus in the zoom is indicated with a white dashed box. Asterisk indicates distal tip of the gonad. Bar, 5 μm. (F) Western blot analysis of whole worm lysates from control and *mrg-1* RNAi-treated worms without *che-1* overexpression using the indicated antibodies against specific histone modifications. Detection of histone H3 serves as the loading control. (G) Immunostaining of gonads from control and *mrg-1* RNAi-treated worms using the indicated antibodies against specific histone modifications. Staining for LIN-53 (shown as overlay with DAPI) serves as a control for staining efficiency. Bar, 5 μm.

RNAi against *mrg-1* permits germ cells to adopt neuronal characteristics by changing their morphological appearance, and turning on expression of neuronal genes upon induction of *che-1* overexpression. However, it is possible that germ cell characteristics are still preserved in cells expressing *gcy-5p*::*gfp*. To address this, we assessed expression of the germline-specific *pie-1* reporter (*pie-1*::*RFP*::*histone*) (Figure 4, A and B) and immunostained for germline-specific P granules. Both germ cell–specific characteristics are lost in GFP-positive cells upon conversion (Figure 4, A and B). Hence, adoption of neuronal gene expression accompanied by the loss of germ cell fate features further substantiates the notion that germ cells can be reprogrammed into ASE neuron-like cells upon RNAi against *mrg-1*. Notably, we did not observe expression of genes that belong to other neuronal fates such as interneurons or GABAergic motor neurons (Figure S4A), indicating the specificity of ASE neuron fate induction in reprogrammed germ cells by CHE-1. Hence, germ cells that fail to show *gcy-5p*::*gfp* but have lost germ cell characteristics may not express other ectopic cell fates.

### MRG-1 safeguards germ cell identity independently of LIN-53 and PRC2

We wondered whether the germ cell conversion in *mrg-1* RNAi animals might be due to a loss of the previously identified germ cell reprogramming barrier LIN-53 (Tursun *et al.* 2011; Kolundzic *et al.* 2018b). LIN-53 acts with the PRC2, which represses chromatin by catalyzing methylation of histone H3K27, to counteract CHE-1–induced germ cell conversion (Patel *et al.* 2012). We examined whether *mrg-1* depletion affects *lin-53* expression in the germline. However, *mrg-1*–depleted animals, with or without *che-1* overexpression, do not show obvious alterations of LIN-53 levels in the germline as assessed by immunostainings (Figure 4D). Interestingly, MRG-1 proteins only partially colocalize with LIN-53 in germ cell nuclei (Figure 4E), indicating that both proteins might have little functional overlap to protect the germline. Furthermore, RNAi against *lin-53* and other PRC2 subunits causes global loss of the PRC2-mediated histone modification H3K27me3 in the germline (Patel *et al.* 2012), which we did not observe upon *mrg-1* depletion in whole worms (Figure 4F) or specifically in the germline (Figure 4G). Overall, these findings indicate that *mrg-1* safeguards germ cells through mechanisms that are not related to PRC2-mediated regulation. Notably, *mrg-1* RNAi animals show a slight increase of the constitutive heterochromatin mark H3K9me3, as well as an increase of H3K14ac (Figure 4, F and G), which has been implicated in DNA damage checkpoints in yeast (Wang *et al.* 2012). However, it is unknown which genomic DNA-binding sites are occupied by MRG-1, and whether MRG-1 is directly linked to regulating histone modifications.

### DNA-binding sites of MRG-1 in the germline and soma

**Figure 5:**
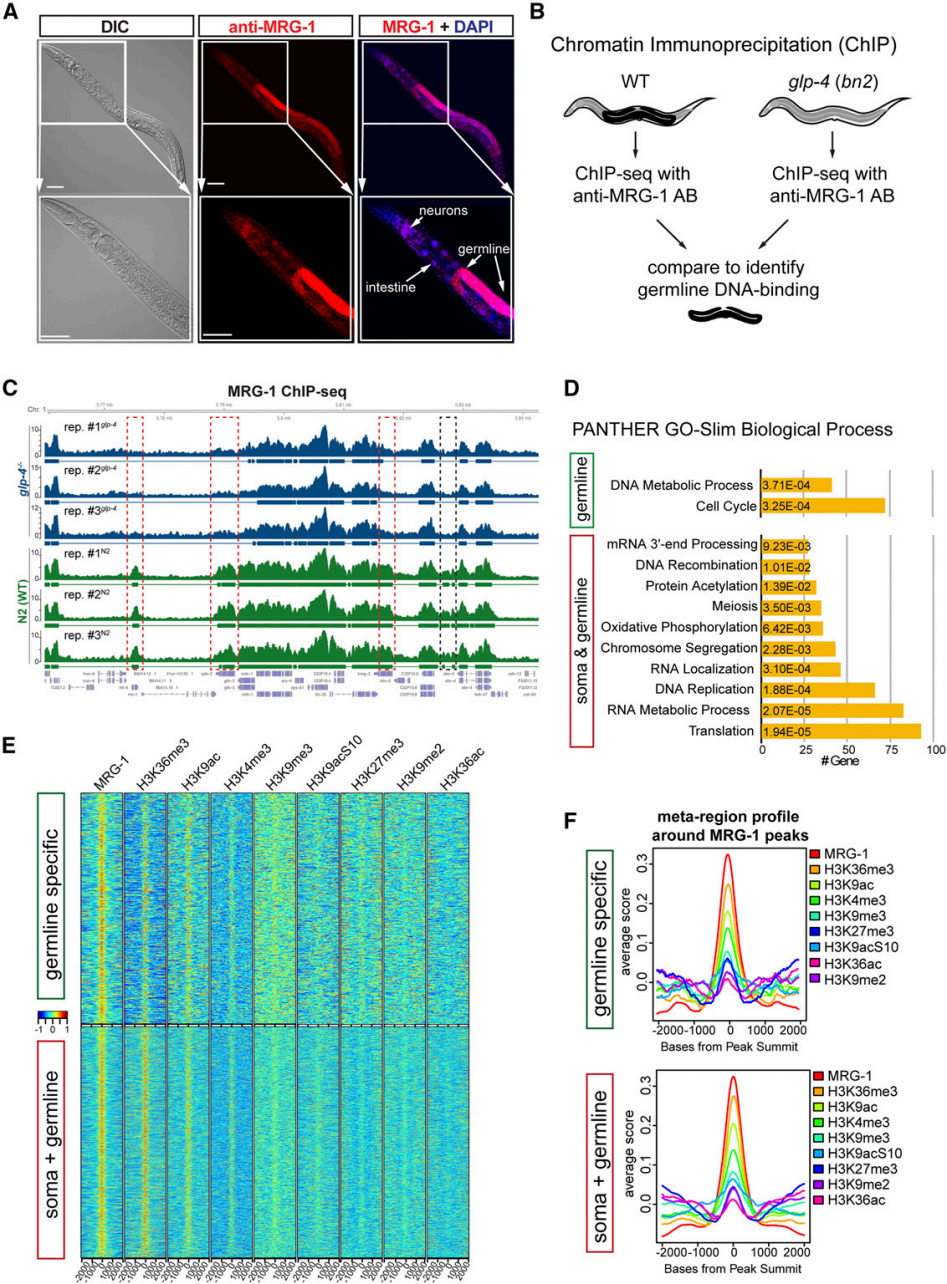
MRG-1 ChIP-seq in soma *vs.* germline. (A) Immunostaining of wild-type young adult hermaphrodite with MRG-1 antibody. MRG-1 proteins are detectable in the germline and predominantly in neurons and the intestine. Costaining for LIN-53 is shown in Figure S5. Bar, 20 μm. (B) To distinguish genome-wide DNA-bindings sites in the soma and germline, MRG-1 ChIP-seq was performed in wild-type and the germline-lacking *glp-4 (bn2)* background. (C) Browser shot of a representative genomic region on chromosome 1, illustrating MRG-1 ChIP-seq peaks from all three replicates for wild-type and *glp-4 (bn2)* background. Red boxes mark genes that cannot be detected as MRG-1 bound in the germline-less background. Black box marks an intergenic region that cannot be detected as MRG-1 bound in the germline-less background. (D) Gene Ontology term analysis using PANTHER Mi *et al.*, 2013 of gene loci bound by MRG-1. (E) Heatmaps showing enrichment of histone modifications at MRG-1 ChIP-seq peaks overlapping in at least two of the wild-type samples (5141) and peaks that were only identified in wild-type but not in *glp-4(bn2)* background (521). The Bar is the scaled centered peak score. (F) Meta-region profile showing the overall distribution averaged over all peaks for MRG-1 ChIP-seq peaks overlapping in at least two of the wild-type samples and peaks which were only identified in wild-type but not in the *glp-4(bn2)* background.

To provide clues as to how MRG-1 contributes to safeguarding germ cells against reprogramming, we sought to reveal the genome-wide DNA-binding patterns of MRG-1 by performing ChIP-seq. Importantly, MRG-1 proteins can be detected in the germline, as well as in somatic cells including neurons and intestinal cells (Figure 5A and Figure S4B). To distinguish between germline-specific and somatic MRG-1 genome binding sites, we used wild-type animals and *glp-4* temperature-sensitive mutants (*bn2*) that lose the germline when grown at 25° (Beanan and Strome 1992) (Figure 5B). Subsequent comparison of DNA-binding patterns from these two backgrounds provided information about MRG-1 DNA-binding sites in all tissues *vs.* the germline in a highly reproducible manner (Figure 5C, Figure S5, A and B, and Table S3).

Overall we identified ∼6723 DNA-binding sites for MRG-1 in the genome of the WT (N2) background (Figure 5A and Table S3), of which 1183 are differential peaks when compared to the germline-less *glp-4* background (Figure S5B and Table S3). Gene-set enrichment analysis using PANTHER (Mi *et al.* 2013) revealed that MRG-1 target genes, in the soma and germline, are predominantly involved in the regulation of translation, RNA processing, and DNA replication and recombination (Figure 5D). Genes that are bound by MRG-1 exclusively in the germline regulate cell cycle and contribute to DNA metabolic processes (Figure 5D). These enriched biological processes of MRG-1 targets concur with findings from previous studies that have implicated MRG-1 and MRG15 in genome integrity, DNA recombination, messenger RNA processing, and germline regulation and proliferation (Takasaki *et al.* 2007; Luco *et al.* 2010; Dombecki *et al.* 2011; Xu *et al.* 2012; Gupta *et al.* 2015; Iwamori *et al.* 2016). Furthermore, human MRG15 associates with the specific histone modification H3K36me3 (Zhang *et al.* 2006; Luco *et al.* 2010), which has also been proposed for MRG-1 in conjunction with the SET domain-containing H3K36 methyltransferase MES-4 in *C. elegans* (Rechtsteiner *et al.* 2010). To test which histone modifications are enriched at MRG-1 DNA-binding sites in *C. elegans*, we made use of available modENCODE data sets (Gerstein *et al.* 2010) and analyzed the overlap of MRG-1 peaks with different histone modifications (Figure 5, E and F and Figure S6, A and B). Soma and germline-shared MRG-1 binding sites correlate predominantly with H3K36me3, H3K9ac, and H3K4me3, while association with genomic loci carrying the repressive histone modifications H3K9me3 or H3K27me3 is rather low (Figure 5, E and F and Figure S6, B and C). The correlation pattern does not change drastically for germline-exclusive MRG-1-binding sites, except for H3K27me3-carrying loci, which become slightly more pronounced (Figure 5, E and F and Figure S6, B and C). Such genes bound by MRG-1 carrying H3K9me3 or H3K27me3 may be direct targets of MRG-1 for repression (Table S4). Overall, MRG-1 predominantly binds genomic loci carrying H3K36me3, H3K9ac, and H3K4me3, which are histone modifications that mark active genes [reviewed by Bannister and Kouzarides (2011), Tessarz and Kouzarides (2014), Hyun *et al.* (2017)], suggesting that MRG-1 might protect germ cells against conversion to neurons not by acting as a repressive chromatin regulator, but by maintaining the genomic integrity and expression of germline components, as previously demonstrated (Wu *et al.* 2012; Xu *et al.* 2012).

### Protein interaction network of MRG-1

**Figure 6:**
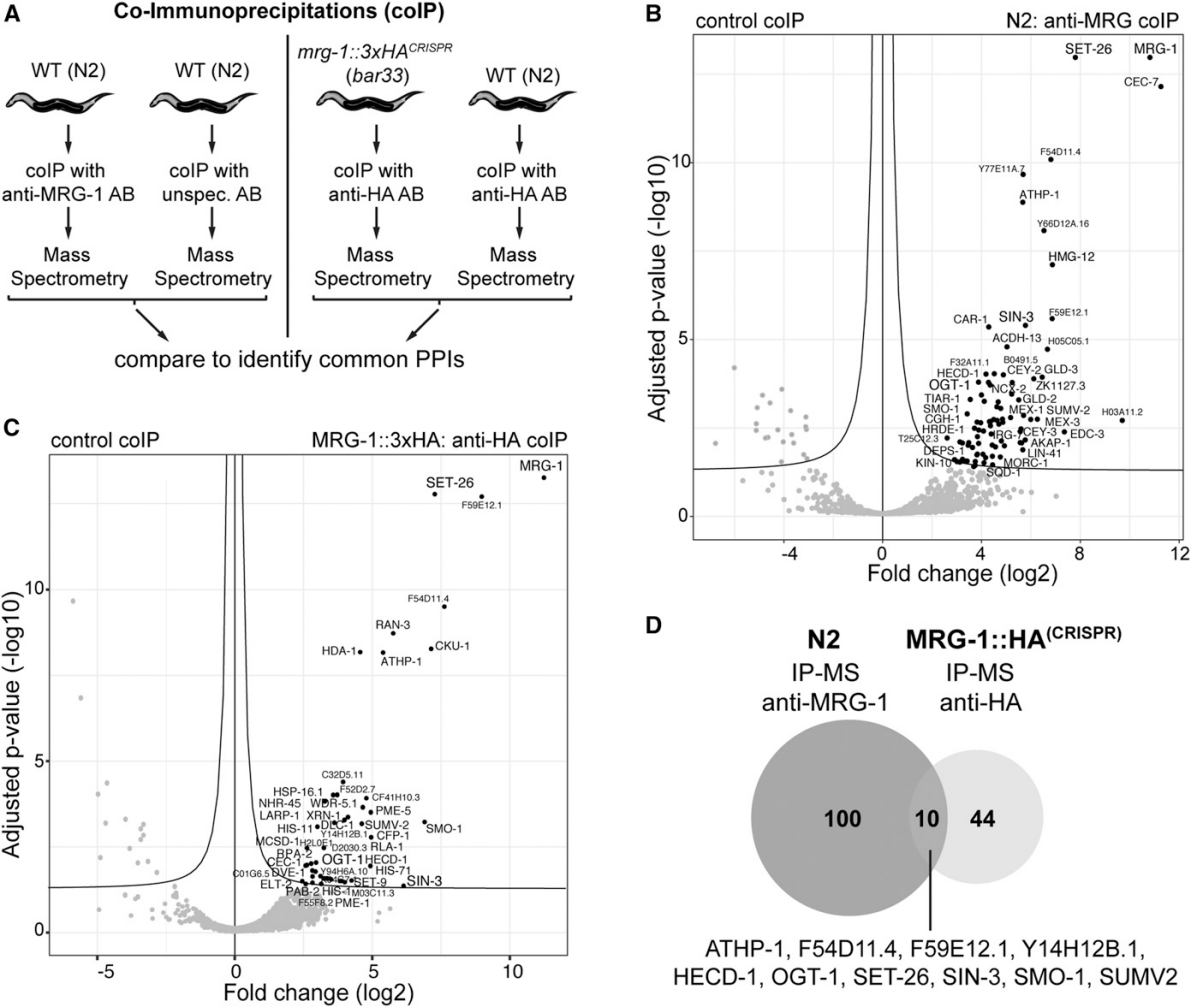
Protein-protein interactions of MRG-1. (A) Co-immunoprecipitations (co-IPs) with subsequent mass spectrometry (IP-MS) to assess MRG-1 protein interactions. Wild type (N2) was used with anti-MRG-1 and unspecific antibodies as control. Additionally, a strain carrying a CRISPR/Cas9-mediated 3xHA knock-in at the *mrg-1* locus (*mrg-1*::*3xHA*^CRISPR^) was used for co-IPs, with HA antibodies also from N2 worms (no HA tag) as the corresponding negative control. (B and C) Volcano plots showing statistically significant enrichment of coprecipitated proteins. Statistics: *t*-test, adjusted *P*-value of 0.05 as false discovery rate cut-off. (D) Common enriched proteins by anti-MRG-1 and anti-HA antibodies.

Next, we asked whether MRG-1 protects the germline fate in complex with other proteins. We therefore investigated the protein interaction network of MRG-1 by performing IP-MS. IP-MS was performed in the wild-type background using anti–MRG-1 antibodies and, to reduce the identification of false-positive protein interactions, we also generated a CRISPR/Cas9-mediated 3xHA knock-in to perform IP-MS using HA antibodies, and compared enriched proteins from both experiments (Figure 6A). IP-MS using anti–MRG-1 yielded 100 enriched proteins, while IP-MS with HA antibodies in the *mrg-1*::*3xHA^CRISPR^* strain yielded 44 proteins (Figure 6, B and C and Table S5). Proteins enriched in both IP-MS experiments, which we considered as the most reliable interacting proteins, were ATHP-1, F54D11.4, F59E12.1, Y14H12B.1, HECD-1, OGT-1, SET-26, SIN-3, SMO-1, and SUMV2 (Figure 6D, Figure S7, and Table S5).

Interestingly, components of the repressive chromatin regulator PRC2 or the SET domain-containing H3K36 methyltransferase MES-4 could not be detected in any of the IP-MS experiments. In contrast, the SET domain protein SET-26 co-immunoprecipitated as one of the strongest interacting proteins overall (Figure 6, B and C and Table S5). Notably, SET-26 has H3K9 methylation activity (Greer *et al.* 2014) and could therefore mediate the chromatin silencing and gene repression activities of MRG-1. Furthermore, the SIN3 family histone deacetylase (HDAC) protein SIN-3 is involved in chromatin repression (Choy *et al.* 2007; Checchi and Engebrecht 2011) and is predicted to associate with another newly identified MRG-1-interacting protein: the β-linked N-acetylglucosamine (O-GlcNAc) transferase OGT-1 (Yang *et al.* 2002; Choy *et al.* 2007; She *et al.* 2009). OGT-1 is the ortholog of the human O-GlcNAc transferase OGT and plays a role in nutrient sensing and insulin signaling pathways, both of which are involved in lifespan regulation in *C. elegans* (Hanover *et al.* 2005; Love *et al.* 2010; Mondoux *et al.* 2011; Radermacher *et al.* 2014). In addition, OGT-1 can be part of histone acetyltransferase-containing protein complexes [Hoe and Nicholas 2014; reviewed by Gambetta and Müller (2015)], suggesting a direct involvement in chromatin regulation. In summary, our IP-MS identified novel MRG-1 interactions and excludes the possibility of direct MRG-1 association with PRC2 or MES-4. Since the newly identified interactors SIN-3, SET-26, and OGT-1 mediate chromatin regulation, they could potentially contribute to MRG-1’s function in protecting the germ cell fate.

### SET-26 and OGT-1 might cooperate with MRG-1 to protect germ cells

**Figure 7:**
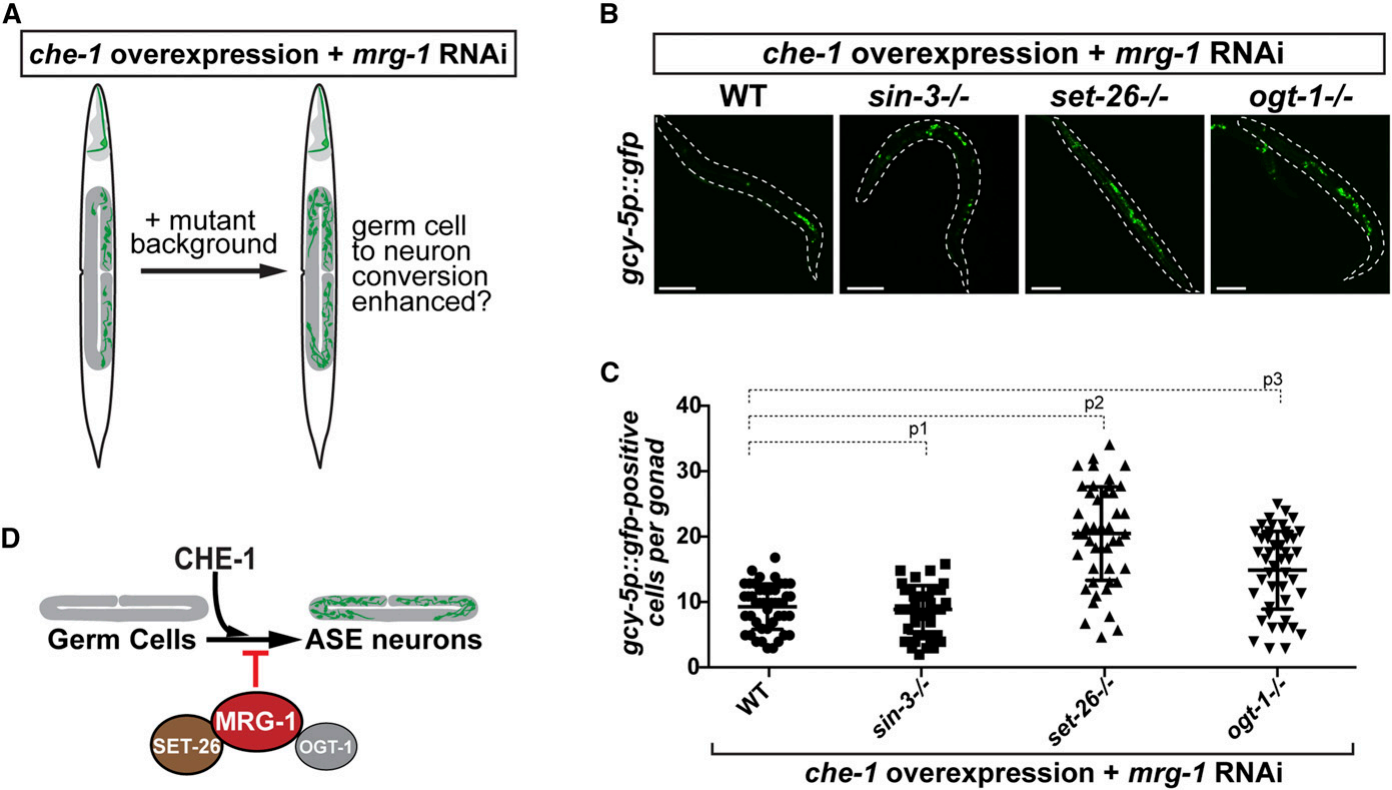
Enhancement of *mrg-1* RNAi-mediated germ cell reprogramming. (A) Rational for testing animal carrying mutations of newly identified MRG-1 interactors for involvement in MRG-1’s role in safeguarding germ cells. (B) The mutant backgrounds of *sin-3 (tm1276)*, *set-26 (tm2467)*, and *ogt-1 (ok430)* were tested for enhancement of *mrg-1* RNAi-mediated germ cell reprogramming. Bar, 20 μm. (C) Quantifications of converted germ cells upon *mrg-1* RNAi in *sin-3 (tm1276)*, *set-26 (tm2467)*, and *ogt-1 (ok430* backgrounds. Number of *gcy-5p*::*gfp*-positive cells in individual gonads were counted. One-way ANOVA multiple comparison: *P*^1^ = 0.7083, *P*^2^ < 0.0001, *P*^3^< 0.0001. (D) Model illustrating that MRG-1 associates with SET-26 and OGT-1 to counteract the conversion of germ cells to ASE neuron-like cells by the Zn-finger TF CHE-1.

To examine whether the protein-protein interactions of SIN-3, SET-26, or OGT-1 with MRG-1 are relevant for MRG-1’s function in protecting the germline fate, we tested whether the mutant backgrounds *sin-3 (tm1276)*, *set-26 (tm2467)*, and *ogt-1 (ok430)* affect the *mrg-1* RNAi-mediated conversion of germ cells into ASE neuron-like cells (Figure 7A). We quantified the number of *gcy-5p*::*gfp*-positive cells in gonads showing germ cell-to-neuron conversion (Figure 7, B and C). While the *sin-3 (tm1276)* mutant background showed no changes in the number of reprogrammed germ cells when compared to the control wild-type background, *set-26 (tm2467)* and *ogt-1 (ok430)* mutations yielded an increase in reprogramming efficiency upon *mrg-1* RNAi. On average, the *set-26 (tm2467)* mutant background allowed an approximately twofold increase in the number of germ cells that convert to neurons, while the quantified increase in the *ogt-1 (ok430)* background is less pronounced at around 1.5-fold (Figure 7, B and C). These observed enhancements in the number of *gcy-5p*::*gfp*-positive cells in the reprogrammed germlines of *mrg-1* RNAi animals suggest that the newly identified interaction of MRG-1 with SET-26 and OGT-1 could be relevant for MRG-1’s role in protecting germ cells from being converted to neurons (Figure 7D).

## Discussion

### Automated screening with solid-phase RNAi identified new safeguarding factors

To identify factors that play a role in protecting *C. elegans* cells against reprogramming to neurons, we aimed to setup an automated RNAi screening system. Because we encountered a decrease in germ cell conversion when depleting LIN-53 in liquid *vs.* solid culture, we established an automated screening pipeline allowing worm growth on solid RNAi media. Compared to a previous procedure described by the Ewbank group (Squiban *et al.* 2012), our setup bypasses the requirement for manual transfer of animals from solid RNAi medium to the screening unit. Using our complemented Chromatin 2.0 library for a P0 RNAi screen, we identified 10 factors that prevent ectopic induction of *gcy-5p*::*gfp*. Interestingly, ectopic *gcy-5p*::*gfp* induction occurs in distinct tissues suggesting that different cell identities are protected by specific mechanisms. The investigation of such tissue-specific mechanisms can provide further knowledge about the different modes of cell fate maintenance and protection.

### MRG-1 safeguards the germ cell fate independently of PRC2

We focused on examining MRG-1, which allowed a germ cell-to-neuron conversion upon RNAi, as recently described for components of the PRC2 complex. MRG-1 is orthologous to the mammalian Mortality Factor 4 Like 1 (MORF4L1), also known as MRG15 (Yochum and Ayer 2002; Olgun *et al.* 2005; Takasaki *et al.* 2007; Chen *et al.* 2009). We used RNAi to deplete *mrg-1* because animals carrying the *hsp*::*che-1* (*otIs305*) transgene in combination with balanced *mrg-1* mutants (*ok1262*, *qa6200*, and *tm1227*) were not viable. Since we previously showed that in *lin-53* and *mes-2* (PRC2), homozygous mutants (M+Z−) derived from heterozygous mothers could not recapitulate the RNAi-based germ cell reprogramming due to maternal rescue effects (Tursun *et al.* 2011; Patel *et al.* 2012), we speculate that such maternal rescue is also a likely scenario for *mrg-1* (M+Z−) mutants.

Recently, MRG-1 was shown to be involved in regulating gene expression and antagonizing the germline fate in the intestine, as well as differentiating the mitotic germline from meiotic and mature germ cells in *C. elegans* (Takasaki *et al.* 2007; Petrella *et al.* 2011; Gupta *et al.* 2015). Additionally, an interplay of MRG-1 with the PRC2 complex during germ cell development has been proposed (Rechtsteiner *et al.* 2010). Since our ChIP-seq results revealed MRG-1 binding to H3K27me3-carrying genes, a cooperation of MRG-1 with PRC2 and LIN-53 could be possible. However, other findings argue against interplay between MRG-1 and PRC2/LIN-53 in protecting germ cells against reprogramming. MRG-1 showed very limited colocalization with LIN-53 in germ cell nuclei and we could not detect protein-protein interactions with LIN-53 or any of the PRC2 subunits. Furthermore, loss of *lin-53* and other PRC2 subunits causes global H3K27me3 decrease in the germline (Patel *et al.* 2012), which we did not observe upon *mrg-1* depletion. Hence, our findings suggest that MRG-1 safeguards the germ cell fate independently of PRC2 and LIN-53.

### MRG-1 and the H3K36 methyltransferase MES-4 do not physically interact

Because MRG-1 preferentially associates with DNA loci that carry the histone modification H3K36me3, which is catalyzed by MES-4 (Rechtsteiner *et al.* 2010), we hypothesized that MRG-1 and MES-4 might directly interact which each other. Interestingly, the MRG-1 ortholog Mrg15 in *Drosophila* promotes the methylation of H3K36 by reinforcing chromatin association of the methyltransferase Ash1 (Huang *et al.* 2017), and such an interaction during chromatin recruitment has also been proposed for MRG-1 and MES-4 (Rechtsteiner *et al.* 2010). However, we did not detect an interaction of MES-4 with MRG-1 by any of the IP-MS experiments. Hence, it remains to be determined whether MRG-1 and MES-4 may indirectly cooperate in protecting the germ cell fate. Previously, MRG-1 was found to be required for X-chromosomal silencing, and an indirect mechanism for its repressive effect has been suggested (Takasaki *et al.* 2007). It is possible that MRG-1 contributes to repressing chromatin in an indirect manner similar to MES-4, by helping to focus PRC2-mediated methylation of H3K27 (Gaydos *et al.* 2012). Since MRG-1 depletion does not lead to a detectable loss of H3K36me3, we speculate that the genomic distribution of H3K36me3 might be altered upon *mrg-1* RNAi that, in turn, could affect gene repression.

### MRG-1 binds to genes that regulate metabolism, replication, and cell cycle

Overall, genes bound by MRG-1 are enriched for functions in DNA metabolism, replication, and cell cycle, as well as chromosome segregation, which is in line with recent findings that MRG-1 and its ortholog MRG15 are implicated in chromosomal break repair and homologous pairing (Garcia *et al.* 2007; Hayakawa *et al.* 2010; Dombecki *et al.* 2011). Therefore, it is possible that a lack of MRG-1 leads to DNA damage thereby causing the observed increase of H3K14ac in the *mrg-1* RNAi germline, an effect that has previously been shown in yeast and mouse (Kim *et al.* 2008; Wang *et al.* 2012). An increase in H3K14ac might lead to a decreased efficiency of H3K9 methylation, as previously suggested (Alvarez *et al.* 2011), which results in lowering or redistributing repressive chromatin marks in the germline. Nevertheless, how such negative cross-talk between these histone modifications might be regulated remains to be determined.

### MRG-1 associates with different chromatin-regulating factors

The interaction of MRG-1 with different chromatin-regulating complexes could provide clues as to how MRG-1 functions at the molecular level. As shown for its mammalian ortholog MRG15 (Yochum and Ayer 2002; Doyon *et al.* 2004; Chen *et al.* 2009), we found an interaction of MRG-1 with SIN-3, the ortholog of the mSin3A HDAC subunit. Notably, we also identified the ortholog of the human O-GlcNAc transferase (OGT) OGT-1 as a novel MRG-1-interacting protein. OGT has been shown to interact with Sin3A in mammalian cells and is thereby being recruited to promoters of repressed genes (Yang *et al.* 2002), indicating that MRG-1 might form a complex with an OGT-1-containing Sin3 HDAC. Yet, the *Drosophila* ortholog of OGT-1 was initially identified as a member of Polycomb group (PcG) class proteins, which are repressive chromatin regulators (Ingham 1984). Additional studies suggest that OGT-1 can be part of histone acetyltransferase-containing protein complexes [Hoe and Nicholas 2014; reviewed by Gambetta and Müller (2015)]. We identified the H3K9 methyltransferase SET-26 as one of the most consistent MRG-1–interacting proteins (Greer *et al.* 2014). SET-26 plays a role in the transgenerational sterility of *spr-5* mutants and a *set-26* mutation suppresses developmental defects seen in animals lacking the NuRD and MEC complex subunit LET-418 (Mi2) (Greer *et al.* 2014; Erdelyi *et al.* 2017). Interestingly, we observed a slight increase in H3K9 methylation in *mrg-1* RNAi animals, which has previously been reported for *mrg-1* mutants (Xu *et al.* 2012). This effect is counterintuitive because we assumed that *mrg-1* depletion causes more open chromatin based on the observed permissiveness for reprogramming. Furthermore, association of MRG-1 with H3K9-methylated genomic sites, although to a limited degree, indicated that SET-26 might be directly involved in MRG-1’s role as a barrier for germ cell reprogramming. Interestingly, the *set-26* mutant background significantly increased germ cell reprogramming upon *mrg-1* RNAi, while a modest enhancement could also be observed for the *ogt-1*, but not for *sin-3*, mutant background. Based on the high reproducibility of the protein interaction data, we therefore suggest that MRG-1 forms a complex with SET-26 and OGT-1 to counteract the conversion of germ cells to neuron-like cells. However, we cannot exclude the possibility that SET-26 and OGT-1 contribute to germ cell protection also in parallel to their interaction with MRG-1.

Overall, we demonstrate the value of enhanced RNAi screens for identifying factors that safeguard cellular identities and the use of *C. elegans* as a gene discovery tool. In light of recent findings illustrating conservation of reprogramming barriers from worms to mammalian tissues (Tursun *et al.* 2011; Cheloufi *et al.* 2015), further genetic screens using different cell fate-inducing backgrounds in *C. elegans* have the potential to identify other context-specific factors that regulate cellular reprogramming, both in *C. elegans* and other species.
